# Biomedical Text Mining and Its Applications

**DOI:** 10.1371/journal.pcbi.1000597

**Published:** 2009-12-24

**Authors:** Raul Rodriguez-Esteban

**Affiliations:** Pfizer Research Technology Center, Cambridge, Massachusetts, United States of America; Whitehead Institute, United States of America

## Introduction

This tutorial is intended for biologists and computational biologists interested in adding text mining tools to their bioinformatics toolbox. As an illustrative example, the tutorial examines the relationship between progressive multifocal leukoencephalopathy (PML) and antibodies. Recent cases of PML have been associated to the administration of some monoclonal antibodies such as efalizumab [1]. Those interested in a further introduction to text mining may also want to read other reviews [2]–[4].

Understanding large amounts of text with the aid of a computer is harder than simply equipping a computer with a grammar and a dictionary. A computer, like a human, needs certain specialized knowledge in order to understand text. The scientific field that is dedicated to train computers with the right knowledge for this task (among other tasks) is called natural language processing (NLP). Biomedical text mining (henceforth, text mining) is the subfield that deals with text that comes from biology, medicine, and chemistry (henceforth, biomedical text). Another popular name is BioNLP, which some practitioners use as synonymous with text mining.

Biomedical text is not a homogeneous realm [5]. Medical records are written differently from scientific articles, sequence annotations, or public health guidelines. Moreover, local dialects are not uncommon [6]. For example, medical centers develop their own jargons and laboratories create their idiosyncratic protein nomenclatures. This variability means, in practice, that text mining applications are tailored to specific types of text. In particular, for reasons of availability and cost, many are designed for scientific abstracts in English from Medline.

## Main Concepts

### Terms

**Figure 1:**
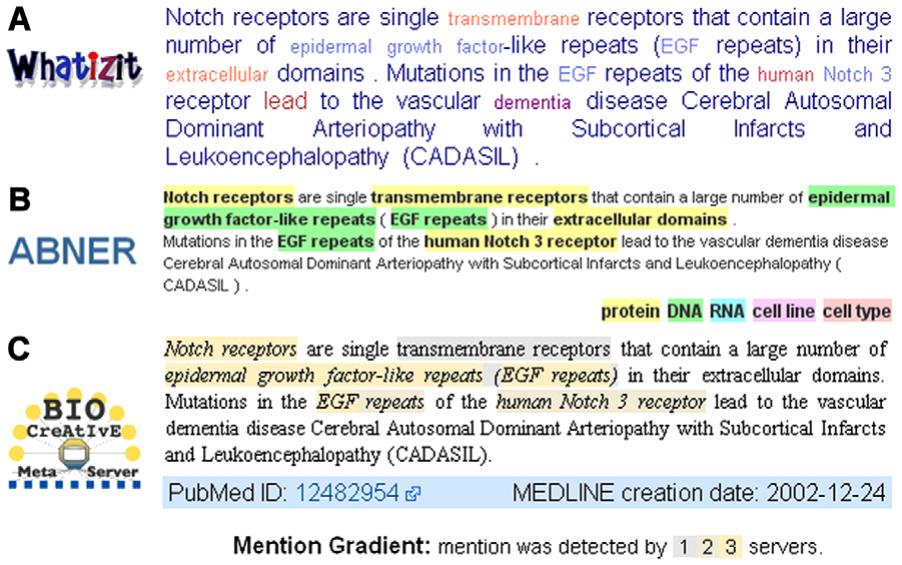
Examples of term recognition. (A) Text marked with protein (blue), disease (crimson), Gene Ontology (bright red), chemical (dark red), and species (red) terms by Whatizit [15] with the *whatizitEBIMedDiseaseChemicals* pipeline. (B) Text marked with protein and cell line terms by ABNER [16]. (C) Protein terms identified by the prototype BIOCreAtIvE metaserver [68]. In the example shown, the metaserver combines the output of systems hosted in three servers.

A *term* is a name used in a specific domain, and a *terminology* is a collection of terms. Terms abound in biomedical text, where they constitute important building blocks. Some examples of terms are the names of cell types, proteins, medical devices, diseases, gene mutations, chemical names, and protein domains [7]. Due to their importance, text miners have worked to design algorithms that recognize terms (see examples in Figure 1). The task of recognizing terms is also called *named entity recognition* in the text mining literature, although this NLP task is broader and goes beyond recognition of terms. Although the concept of term is intuitive (or, perhaps, *because* it is intuitive), terms are hard to define precisely [8]. For example, the text “early progressive multifocal leukoencephalopathy” could possibly refer to any, or all, of these disease terms: “early progressive multifocal leukoencephalopathy,” “progressive multifocal leukoencephalopathy,” “multifocal leukoencephalopathy,” and “leukoencephalopathy.” To overcome such dilemmas, text miners ask experts to identify terms within collections of text such as sets of selected Medline abstracts. These annotations are then used to train a computer by example, so that the computer can emulate the knowledge experts deploy when they read biomedical text. This pedagogical method, “teaching by example,” is a common approach used in many text mining tasks and it is more generally called supervised training. (Alternatively, text miners create rules using expert knowledge.) Thus, text miners rely heavily on collections of text (corpora) that have been annotated by experts (see compilations of corpora: http://www2.informatik.hu-berlin.de/~ hakenber/links/benchmarks.html; http://compbio.uchsc.edu/ccp/corpora/obtaining.shtml). Before beginning a text mining task, it is advisable to limit the scope of the task to a corpus made of a set of documents around the topic of interest. In our case, a PML corpus could comprise all the Medline abstracts that mention the term “progressive multifocal leukoencephalopathy,” because this is an unambiguous term. Another relevant corpus to consider could be the ImmunoTome [9], which is focused on immunology.

Text miners are interested in terminologies that have been built manually. These controlled terminologies have notable roles in biomedicine, for example, the HUGO gene nomenclature, the ICD disease classification, or the Gene Ontology. Many of these terminologies are more than just a flat list of terms. Some include term synonyms (thesauri) or relations between terms (taxonomies, ontologies). For text miners, their usefulness comes from their ability to link to information. Once a text is mapped to one of these terminologies, a bridge is opened between the text and other resources. This usefulness justifies efforts such as the National Library of Medicine's manual mapping of Medline abstracts to the Medical Subject Headings (MeSH) terminology. In our example, MeSH can be used to make the PML corpus more focused by restricting it only to abstracts with the MeSH term “leukoencephalopathy, progressive multifocal.” Controlled terminologies can be used to annotate results from experiments and databases [10]. Text miners attempt to make such mappings automatically. For example, a task called *gene normalization* consists in recognizing names of genes in text and mapping them to their corresponding gene identifiers (e.g., Entrez Gene ID). Thus, using gene normalization it is possible to identify all the abstracts in Medline that mention a given gene from Entrez Gene [11].

Because there are many controlled terminologies, some terminologies have been created to map between them. For example, the BioThesaurus [12] is a compilation of protein synonyms from several terminologies. The Unified Medical Language System (UMLS) [13],[14] is a grand compilation of more than 120 terminologies and close to 4 million terms. Despite UMLS's size, all controlled terminologies are incomplete, because new terms are created too quickly to keep them up to date. Furthermore, all have gaps and areas of emphasis that conflict with the needs of users.

### Tools for Terms

Whatizit [15] is a tool that recognizes several types of terms. It can be accessed through a Web interface, Web services, or a streamed servlet. Abner [16] is a standalone application that recognizes five types of terms: protein, DNA, RNA, cell line, and cell type. More specialized term recognition has been used, for example, for databases such as LSAT [17] for alternative transcripts and PepBank [18] for peptides. Text miners have also used terminologies to enrich PubMed's search capabilities. Some recent search engines are semedico [19], novo|seek [20], and GoPubMed/GoGene [21],[22].

### Relationships

After recognizing terms, the natural next step is to look for relationships between terms. The simplest method to identify relationships is using the *co-occurrence* assumption: terms that appear in the same texts tend to be related. For example, if a protein is mentioned often in the same abstracts as a disease, it is reasonable to hypothesize that the protein is involved in some aspect of the disease. The degree of co-occurrence can be quantified statistically to rank and eliminate statistically weak co-occurrences (see Box 1). An example using GoGene [22] can illustrate the use of simple co-occurrence, MeSH terms, and gene normalization. The query *“leukoencephalopathy, progressive multifocal”[mh]* in GoGene returns all the genes mentioned in Medline abstracts annotated with the MeSH term for PML. The genes that appear most often are likely to be related to PML. Those that appear disproportionately more often for PML than for other diseases are likely to be more specific to PML.

**Box 1. The strength of a relationship.** The confidence in a fact that comes from text can be qualified by the level of certainty of the assertion where the fact was found or by the strength of the evidence pointed [71]. Since facts do not stand alone, this confidence depends also on the fact's consistency with related facts [72]. In the case of co-occurrence of two terms *t_1_* and *t_2_*, the simplest confidence metric is the count *c* of texts that include both terms, 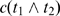 (for a PPI example, see [73]). This measure can be normalized by the possibility of random co-occurrences due to the sheer popularity of one or both terms. For example,
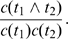

Pointwise mutual information (PMI) is similarly derived as
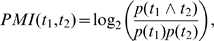
where 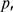, in this case, is 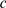 divided by the total number of texts. More generally, different measures can be drawn from the 2×2 contingency table that encompasses the counts of texts that include the two terms, 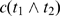, only one term (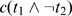 and 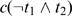), and none, 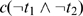. Using this contingency table, Medgene [32] compared the merit of different statistical measures for gene-disease associations such as chi-square analysis, Fisher's exact probabilities, relative risk of gene, and relative risk of disease. More heuristic methods have been devised that use manually adjusted weights for different types of co-occurrence [36].

Better evidence than co-occurrence comes from relationships that are described explicitly [23]. For example, the sentence “We describe a *PML* in a 67-year-old woman with a destructive polyarthritis associated with *anti-JO1 antibodies* treated with corticosteroids” [24] describes an explicit link between PML and anti-JO1 antibodies. We can simplify this relationship into a triplet of two terms and a verb: PML *is associated with* anti-JO1 antibodies. To create the triplet, the verb can be identified with the aid of a part-of-speech (POS) tagger. An example of a POS tagger for biomedical text is MedPost [25]. This triplet representation is powerful due to its simplicity, but it omits crucial details from the original article, such as the fact that the evidence comes from a clinical case study.

**Figure 2:**
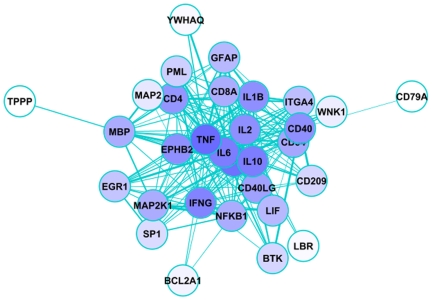
Example of text-mined PPI network. The nodes are proteins identified using the query: *“leukoencephalopathy, progressive multifocal”[mh] antibody[pubmed]* in GoGene [22]. The query retrieves gene symbols mapped to PubMed abstracts that include the keyword *antibody* and the MeSH term *leukoencephalopathy, progressive multifocal* (PML). The gene list was exported to SIF format and the gene symbols extracted and used to query PPI using iHOP Web services [69]. Only those iHOP interactions with at least two co-occurrences and confidence above zero were considered. The network was plotted using Cytoscape [70]. The node color is based on the number of interactions (node degree).

A heavily studied area in text mining concerns the relationships known as protein-protein interactions (PPI). Using the triplet representation, PPI can be depicted as network graphs with the proteins as nodes and the verbs as edges (see Figure 2). When analyzing text-mined interaction networks, it is important to understand the information that underpins them. For example, interactions can be direct (physical) or indirect, depending on the verb (examples of direct verbs are *to bind*, *to stabilize*, *to phosphorylate*; examples of indirect verbs are *to induce*, *to trigger*, *to block*) [26]. The different nature of the protein interactions described in the literature reflects in part the experimental methodology employed and the nature of the interaction itself. A common way to capture the textual variations is by exhaustively identifying all the patterns that appear and writing a set of rules that capture them [27],[28]. For example, a simple pattern to capture phosphorylations might involve, sequentially, a kinase name, a form of the verb *to phosphorylate*, and a substrate name [29],[30].

### Tools for Relationships

To see co-occurrence in action, try FACTA [31]. MedGene and BioGene [32],[33] use co-occurrence for gene prioritization. Gene prioritization tools such as Endeavour [34] and G2D [35] use text as well as other data sources. PolySearch [36] uses heuristic weighting of different co-occurrence measures and includes a detailed guide to implementation and vocabularies. Anni [37] uses textual profiles instead of co-occurrence to measure relationship between terms. For PPI, iHOP [38] is the most popular tool. RLIMS-P [30] uses linguistic patterns to detect the kinase, substrate, and phosphosite in a phosphorylation. E3Miner [39] detects ubiquitinations, including contextual information.

### Discovery

Besides finding relationships, text miners are also interested in *discovering* relationships. Due to the size of the literature, scientists miss links between their work and other, related work. Swanson called these links “undiscovered public knowledge.” In a classic example he found by careful reading 11 links between magnesium and migraine that had been neglected [40]. One method to discover relationships is based on transitive inference [41]. Simply stated, if A is linked to B, and B is linked to C, then there is a chance that A is linked to C. PPI networks are, at the core, an example of transitive inference. Arrowsmith [42] is a basic discovery tool that compares two literature sets to find links between them. Applying Arrowsmith to the literature for PML and antibodies yields the immunomodulator tacrolimus, a calcineurin inhibitor, among the top hits. Tacrolimus affects the production of several proteins depicted in Figure 2, such as IL-2.

### Quality

The most common measure of output quality in text mining is the F-measure, which is the harmonic mean of two other measures, precision and recall. These three measures can be described with the analogy of searching for needles in a haystack. After a manual search of a haystack, our hands end up full with valuable needles but also with some useless straws. Recall is based on the number of needles found. High recall means that we have found most of the needles for which we were looking. Precision, however, is based on the number of both needles and straws. High precision means that we have retrieved far more needles than straws. Both high precision and high recall are desirable, and a high F-measure reflects both because it is the harmonic mean. Optimizing the F-measure of a text mining application is often different from optimizing the accuracy, because there are usually few needles and large amounts of hay in the haystack. An application that identifies the whole haystack as being only hay is quite accurate but misses all the needles.

It is important to ponder over the way an application has been evaluated before assessing its F-measure [43], and especially to consider how realistic the evaluation was. The F-measure is not an absolute value. The larger a haystack is, the more difficult it is to find needles. In other words, a low F-measure might reflect a harder task, not a worse application. Moreover, text mined applications may perform differently in different types of text and this may be reflected in lower F-measures than advertised. When the F-measure attainable is not high enough, one solution is to use text mining as a filter. A filter needs high recall, but only moderate precision, to reduce the amount of hay without affecting the needles. Filtering with text mining is used as a preliminary step in databases such as MINT [44], DIP [45], and BIND [46]. Filtering is followed by human *curation*, which involves the review and assessment of results to reduce hay and, hopefully, provide feedback to improve the filtering. The feedback loop between text mining and curation can have an incremental positive impact in output results [47].

### Comprehensiveness

Doing comprehensive text mining means considering all sources of information—Medline and beyond. The abstract conveys an article's main findings, but many other pieces of information are elsewhere in the full text, figures, tables, supplementary information, references, databases, Web sites, and multimedia files. In particular, the full text is critical for information that rarely appears in abstracts, such as experimental measurements. A more comprehensive PML corpus would include full text articles, however despite the surge in open access articles (see the Directory of Open Access Journals, www.doaj.org; [48]), the majority of published articles have access and processing restrictions. PubMed Central [49] is the main source of open access articles, and the specialized search engines BioText [50], Yale Image Finder [51], and Figurome [52] search PubMed Central figures and tables. A search for “progressive multifocal leukoencephalopathy” in the Yale Image Finder yields only one figure, while a search for “PML” yields a large number of hits, most of them not relevant because PML is an ambiguous acronym.

### Text and DNA

Considering text as a sequence of symbols as informative as a protein's DNA sequence is the underlying premise of many text mining tools for bioinformatics. For example, the linguistic similarity between protein corpora (sets of texts built around proteins) correlates with the BLAST score between those same proteins [53]. Text that is used in articles or database annotations to describe a protein can be used for protein clustering and to predict structure [54], subcellular localization, and function [55]. For example, a protein corpus of a protein located in the nucleus uses a vocabulary that is somewhat different from a corpus built around a secreted protein. These vocabulary differences can be used to predict the subcellular localization of a protein of unknown location. One way to measure vocabulary differences is to represent the texts as vectors of word counts. The word counts can be normalized by the size of the text they come from and the vectors compared using, for example, Euclidean distance (for more, see [56]). To reduce vector dimensionality, some words can be grouped using a method called stemming. A simple example of stemming is converting plural nouns into singular form and verbs into infinitive form (a widely used stemming algorithm is the Porter stemmer [57]). Additional simplification can be achieved via tokenization, because some words can be separated into constitutive elements called tokens. In English, however, most words are a single token. An example of a word of two tokens is *don't*.

Text mining applications for bioinformatics [58] include subcellular localization prediction such as Sherloc and Epiloc [59],[60] and protein clustering such as TXTGate [61]. Thus, text mining tools can be used for annotating biological databases in the same fashion other bioinformatics tools are used.

### More Tools

An extensive list of text mining applications is maintained in http://zope.bioinfo.cnio.es/bionlp_tools/ [62]. A growing number of tools are being developed under a standard framework called UIMA, which comprises NLP as well as BioNLP tools [63].

## Conclusion

Text mining tools are increasingly more accessible to biologists and computational biologists and these can often be applied to answer scientific questions in combination with other bioinformatics tools. Getting acquainted with them is a first step towards grasping the possibilities of text mining and towards venturing into the algorithms described in the literature. One way to get started on this path is by looking at examples such as [64]–[67].
